# Frequently Recurrent Takotsubo Syndrome in COPD

**DOI:** 10.1155/2019/6706935

**Published:** 2019-01-09

**Authors:** Juan Vaz, Rikard Berggren, Berne Eriksson

**Affiliations:** ^1^Department of Medicine, Halmstad Hospital, Halmstad, Sweden; ^2^Department of Cardiology, Halmstad Hospital, Halmstad, Sweden; ^3^Krefting Research Centre, Institute of Medicine, University of Gothenburg, Gothenburg, Sweden

## Abstract

Cardiovascular disease is common among patients with chronic obstructive pulmonary disease (COPD). Takotsubo syndrome (TTS) is a transient cardiac disorder that, in its typical form, involves left ventricular dysfunction with apical ballooning and mimics acute coronary syndrome (ACS). “Bronchogenic TTS” has been proposed as a specific form of TTS (during severe acute dyspnea in asthma or COPD) with atypical presentation. Recurrent TTS in COPD seems to be exceptionally rare since only a handful of clinical cases have previously been reported in the literature. Here, we present a unique case of a frequently recurrent TTS during COPD exacerbation in a 70-year-old woman, with at least 4 different episodes of TTS within 5 years. This case report exemplifies the difficulties of the diagnosis of TTS at the onset of acute COPD exacerbation. Potential pathophysiological mechanisms and therapeutic strategies are also briefly discussed.

## 1. Introduction

Cardiovascular disease is common among patients with chronic obstructive pulmonary disease (COPD). More than 3.2 million COPD patients die annually, and in approximately 30% of the cases, cardiovascular disease and its complications are the main causes of death [1, 2]. Despite formerly being referred to as “stress cardiomyopathy,” Takotsubo syndrome (TTS) is a complex transient cardiac disorder that, in its typical form, involves left ventricular dysfunction with apical ballooning, and mimics acute coronary syndrome (ACS) [3, 4]. The prevalence of TTS is unknown, but it is estimated to be 2-3% of all patients with diagnosed ACS [5]. Cumulative incidence of recurrence in TTS has been reported to be approximately 5% at 6 years, and the annual rate of recurrence is only 1-2% [3, 6]. TTS has also been related to severe dyspnea during respiratory disease [7, 8], but the pathophysiological mechanisms remain mostly unknown [9]. “Bronchogenic TTS” has been proposed as a specific form of TTS that occurs during severe acute dyspnea in asthma or COPD, with atypical presentation [10–12]. Recurrent TTS in COPD seems to be exceptionally rare since only a handful of clinical cases have previously been reported in the literature [13, 14]. Here, we present a unique case of frequently recurrent TTS during COPD exacerbation with at least 4 different episodes of TTS.

## 2. Case Presentation

At first medical contact (FMC), a 70-year-old Caucasian woman who admitted to be a heavy smoker and was slightly overweight, presented to the emergency room (ER) with severe dyspnea. Her past medical history, medications, and background information are summarized in Table 1.

Hypoxia with pulse oxygen saturation < 85%, tachypnea, tachycardia, and hypertension were present. Signs of infection, cyanosis, and peripheral edema were absent. Clinical examination revealed expiratory wheezes and prolonged expiration. Chest pain and electrocardiogram (ECG) abnormalities were absent. Chest radiography exhibited bilateral flattening of the diaphragm but no pulmonary infiltrates or pneumothorax. White blood cell count (WBC) and C-reactive protein (CRP) were mainly normal, but troponin T (TPNT) level was elevated (53 ng/L: normal level, <15 ng/L). The patient was admitted to the hospital, and standard treatment for acute exacerbation of COPD was initiated.

**Figure 1:**
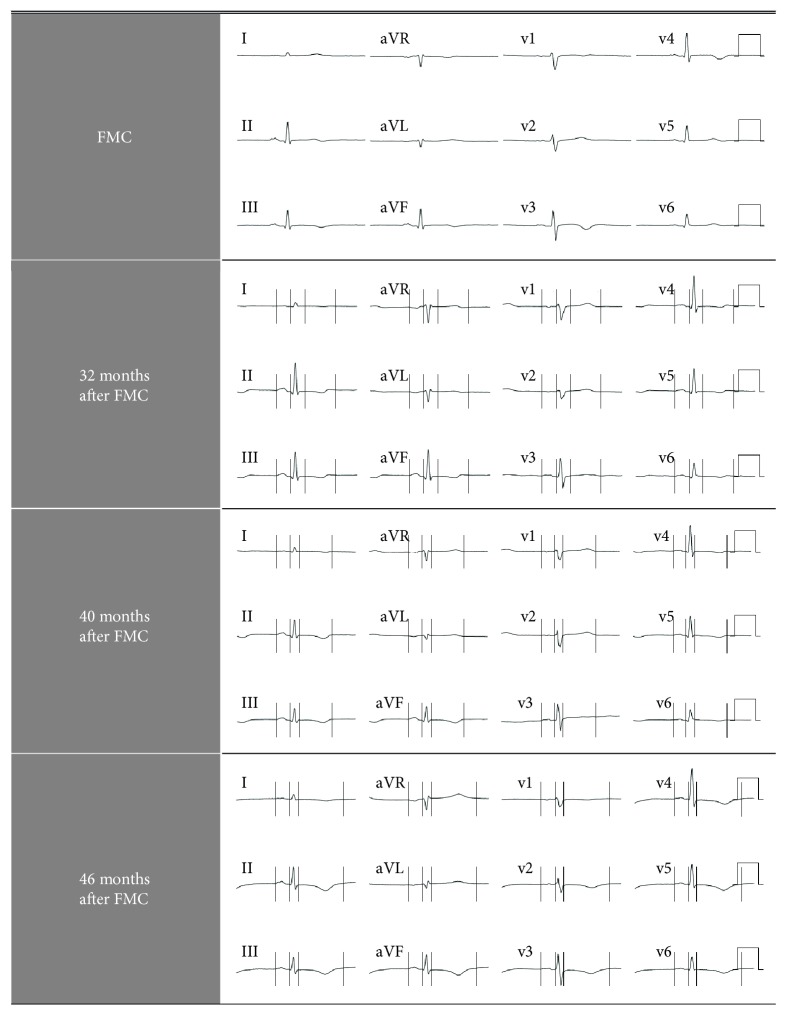
ECG abnormalities in a 70-year-old female COPD patient with recurrent TTS.

Soon after admission, increased dyspnea and vague chest discomfort were observed despite normal pulse oxygen saturation. ECG revealed T-wave inversion in several leads, normal QT interval (Figure 1, FMC), and an increased TPNT level of 108 ng/L. Pulmonary embolism and aortic dissection were outruled via computed tomography (CT). Echocardiography (ECHO) revealed a normal left ventricular ejection factor (LVEF) without dyskinesia, at the time (see under Discussion). Dual antiplatelet therapy (DAPT), in concordance with the existing guidelines of the European Society of Cardiology (ESC) for ACS, was initiated, and coronary angiography was performed. The latter showed no signs of significant stenosis or other pathologies that could explain the patient's symptoms. During the following days, no other episodes of dyspnea or chest pain were registered, ECG and TPNT level returned to normal, and the patient was discharged from the hospital with prescriptions for standard treatment for COPD. Upon the initially assumed absence of pathology in ECHO, the patient was diagnosed with myocarditis and followed up 3 months after FMC with a new ECHO, which was without abnormalities. During the follow-up period, the patient managed to quit smoking.

Almost 32 months after FMC, the patient was again admitted to the hospital. This time she was suffering from exhaustion, dyspnea, and leg swelling for 2 weeks. Bilateral crackles and pitting edema of legs were present. ECG revealed T-wave inversions on several leads without QT interval prolongation (Figure 1). TPNT level was elevated (58 ng/L). Likewise, NT-probrain natriuretic peptide (NT-pro-BNP) level was also abnormal (2088; normal age-adjusted level, <450 pg/mL). CT scan showed no signs of pulmonary embolism, aortic dissection, or pleural effusion. ECHO, at the time, revealed severe left ventricular hypokinesia, most prominent in the inferolateral wall and the septum. LVEF was approximately 30%. Subsequent analysis revealed severe midventricular hypokinesia instead of inferolateral (see under Discussion). DAPT and heparin were given and acute coronary angiography was performed. Once again, no significant pathology was found despite the patient's severe acute heart failure. Also, as during FMC and upon treatment with diuretics and standard COPD medications, dyspnea and chest pain disappeared, ECG returned to normal, and crackles and pitting edema were gone. TPNT levels decreased as well (Table 2). The patient was then diagnosed with non-Q-wave myocardial infarction, and she was prescribed metoprolol, ramipril, and eplerenone. Cardiac magnetic resonance imaging (CMR) was performed 3 weeks after discharge from the hospital. Oddly, no signs of previous myocardial infarction or myocarditis were found, LVEF was calculated to be 55%, and hypokinesia was absent. Subsequent follow-ups confirmed that the patient was feeling well without symptoms of heart failure or angina pectoris. Moreover, NT-pro-BNP level was normal and a new ECHO was completely normal. Treatment with eplerenone was discontinued due to adverse side effects, and after some months, follow-ups were discontinued on the patient's initiative.

Eight months after the second episode (4 months after the last follow-up), the patient was once again hospitalized due to acute COPD exacerbation. Chest pain was denied, but a burning feeling over the back was present. Clinical examination was concomitant with previous episodes, but pitting edema and crackles were absent. The initial ECG at ER was normal except for sinus tachycardia, and cardiac troponin, NT-pro-BNP, CRP, and WBC levels were normal.

During the first hours after admission, severe dyspnea was observed and TPNT levels increased to 244 ng/L. ECG now revealed T-wave inversions similar to those found at previous hospitalizations (Figure 1). A small quantity of pleural effusion was found at CT scan, but pulmonary embolism and aortic dissection were absent. ECHO showed severe left ventricular hypokinesia, most manifest in the apical and midventricular segments. LVEF was estimated at 20%. NT-pro-BNP level increased from the previous normal level to 1000 pg/mL. As chest pain was still absent, treatment with intravenous diuretics was started; the patient's status improved rapidly, but the TPNT level remained unchanged. On day 4, severe chest pain started and the TPNT level increased to 300 ng/L. ECG was still abnormal with unspecific T-wave inversions. DAPT was given, and coronary angiography was once again normal. Astonishingly, soon after coronary angiography, chest pain ceased, ECG and NT-pro-BNP were normal, TPNT levels decreased considerably, and ECHO revealed an LVEF of 50-60% without hypokinesia. She was again diagnosed with myocarditis, and furosemide was prescribed. A follow-up within a month was planned.

Almost 2 weeks after being sent home, the patient returned to the ER with dyspnea and acute chest pain. Crackles were present, but chest radiography was normal. Pitting edema was absent. TPNT level was 107 pg/mL. The patient was again admitted to the hospital, and ECHO performed on the next day was evaluated as normal. At this point, the patient was completely recovered and stated that she had had a rough week with high stress levels. The TPNT level decreased, and since a follow-up was previously planned, she was discharged without new prescriptions. She was diagnosed with unspecific chest pain.

During the succeeding follow-ups, a grade 3 COPD was confirmed via spirometry (FEV1/FVC0.39, FEV1 39% of normal) and her COPD treatment was adjusted. Treatment with metoprolol was substituted with amlodipine, and a new ECHO was normal with an LVEF of 60%.

Six months after past hospitalization (4 months after the last follow-up), the patient was again admitted to the hospital due to severe dyspnea. Chest pain was negated, ECG showed T-wave inversion on leads AVL and I, and TPNT was slightly elevated. ECHO at ER revealed severe generalized left ventricular hypokinesia with midventricular akinesia, but the basal and apical segments were less affected. LVEF was estimated to be less than 20%. On day 2, TPNT level increased to 431 ng/L, NT-pro-BNP level was normal, and T-wave inversions on several leads were observed (Figure 1). During the following days, the patient recovered, ECG returned to normal, and TPNT level decreased considerably. Importantly, chest pain was never observed. ECHO previous to discharging from the hospital showed normal global function with an LVEF of 50% with remaining left basal hypokinesia. Treatment with carvedilol was started since the patient did not tolerated amlodipine, and verapamil was considered contraindicated due to a potential interaction with lithium (see Table 1). During this hospitalization, the patient was given the diagnosis of TTS for the first time. Additionally, a comprehensive review of the patient's previous medical history and saved ECHO images exposed several episodes of TTS (see under Discussion).

Finally, 2 months after her first diagnosed TTS, she was again admitted to the hospital due to dyspnea and elevated TPNT (107 ng/L). ECG was mainly normal. For unknown reasons, an ECHO wasn't performed and the patient was discharged on the first day. Cardiac computed tomography angiography (CCTA) was performed after two months, and no significant stenosis was observed. A previously planned ECHO showed normal LVEF and minor left basal hypokinesia. To date, no new TTS episodes have been observed and concurrently, no new acute COPD exacerbations have been noted.

## 3. Discussion

A case of recurrent TTS with different wall motion patterns in the same patient has previously been reported [15]. “Bronchogenic TTS” has been defined as an atypical form of TTS [10–12], and acute respiratory failure has also been associated to atypical TTS [8]. Our review of our patient's preceding medical data uncovered four probable episodes of atypical TTS. In three of them, our ECHO expert established the presence of midventricular hypokinesia, and in one case, akinesia was established; no signs of right ventricular strain and/or pulmonary hypertension were found (Table 2). Midventricular TTS is a rare form of TTS, found in only 14.6% of the cases [3, 4]. Likewise, in FMC, our ECHO expert found signs of apical hypokinesia but not apical ballooning that might be similar to those found in focal type TTS [3]. In one of the episodes, the quality of the data was insufficient for analysis of ECHO images, and in the last episode, no ECHO was performed. In summary, based on the recently published International Takotsubo Diagnostic Criteria (InterTAK Diagnostic Criteria) [4], International Takotsubo Diagnostic Score (InterTAK Diagnostic Score), and diagnostic algorithm of Takotsubo syndrome [16], we advocate that our patient had had 4 different episodes of atypical TTS (1 focal type TTS and 3 midventricular TTS), the last 3 occurring with high frequency (Table 2). This postulation is supported by the occurrence of new ECG abnormalities (T-wave inversions) combined with transient elevation of cardiac troponins, confirmed transient echocardiographic hypokinesia compatible with TTS, and no evidence of infection, myocarditis (even if the patient was previously diagnosed with myocarditis, there is enough clinical data to dismiss this diagnosis since there is no sign of myocarditis on CMR), or significant coronary artery spasms (coronary angiography and CCTA).

TTS is a puzzling and complex medical condition with a largely unknown pathophysiology, and a comprehensive review of it is beyond the scope of this article. Nevertheless, since TTS is frequently elicited by emotional or physical stressors, sympathetic stimulation has long been proposed as a key factor in TTS [4, 17]. Among others, oxidative stress, infective agents, genetic predisposition, transient coronary artery spasm, and estrogen deficiency appear to be involved in the development of TTS [4, 18, 19].

There are several factors in this case that may have contributed to the high recurrence of TTS observed. Female sex, emotional and physical stresses, no ST-segment depression, and psychiatric disorders are the main risk factors in this case, according to the InterTAK Diagnostic Score [16].

### 3.1. Female Sex and Hormonal Factors

Approximately 90% of TTS patients are postmenopausal women [3], which might support estrogen-deficiency theories [20]. In our patient, bilateral salpingo-oophorectomy was performed approximately 15 years before FMC and she had had no estrogen supplementation (Table 1). Estrogen supplementation might attenuate glucocorticoid and catecholamine responses to mental stress in perimenopausal women [21]. However, there is no evidence of similar responses in postmenopausal women and clear associations between estrogen levels and the development of TTS have not been reported yet. Hypothyroidism might predispose to TTS since 35% of the patients in a single center retrospective study (predominantly elderly female) had a history of hypothyroidism and the use of levothyroxine did not have protective effects [22, 23]. Still, the role of hypothyroidism in TTS is unknown and multicentric prospective studies are lacking.

### 3.2. ECG Changes

The InterTAK Registry reports a 41% T-wave inversion frequency in TTS [3]. Moreover, initial T-wave inversion has been described in atypical TTS due to acute respiratory failure [8] and even in “bronchogenic TTS” [10]. Our patient's initial ECG showed unspecific T-wave inversions in all TTS episodes (Figure 1, Table 2). However, no QT interval prolongation was observed, which otherwise is found in up to 50% of TTS patients at admission [3] and might cause life-threatening ventricular arrhythmias during the subacute phase [16]. Interestingly, in a recent study, no ECG differences were found on admission among patients with TTS due to emotional stress compared to patients with TTS secondary to neurological diseases, physical activities, and medical conditions or procedures, respectively [24].

### 3.3. COPD

In 3 of the episodes (including FMC), the development of TTS was observed during the first hours after hospitalization and after successful respiratory therapy with inhaled drugs. Normally, COPD patients are given high doses of a beta agonist (such as terbutaline or salbutamol) combined with ipratropium. As elevated catecholamine levels seem to play a key role in TTS [4], the additional stimulation of cardiac beta-2-adrenergic receptors might potentiate the development of TTS in patients under extreme stress, such as under hypoxia and acidosis. It has been suggested that epinephrine has a stunning effect on myocardial tissue in TTS due to the induction of beta-2 receptors, which in turn were more frequently found in the cardiac apex during TTS [25]. Thus, the use of a high dose of a beta-2 agonist at the ER might have been important for our patient's TTS development. In one occasion, the patient presented to the ER with manifest heart failure (Table 2). The medical records reveal that the patient had been inhaling high doses of salbutamol and ipratropium prior to hospitalization. Remarkably, ipratropium has also been proposed as an inducer in “bronchogenic TTS” [26]. Still, the use of ipratropium may mainly have favorable effects in “bronchogenic TTS” since it may reduce the need of beta-2-agonist administration. Even if hypothetically promising, the use of beta blockers in TTS is controversial and, at 1 year, there is no evidence of survival benefit for the use of beta blockers [3]. Glucocorticoids, such as prednisolone or betamethasone, are often administrated in high doses during acute COPD exacerbation. There is no known relationship between the use of systemic steroids and TTS. Despite being a stressor, high-dose corticosteroids may have had favorable effects in our patient since (as with ipratropium) this obviously reduced the need for beta-2-agonist administration during the subacute phase. Moreover, the anti-inflammatory effects of cortisone might have relieved the patient's chronic muscle pain, decreasing the underlying stress levels and possibly even the level of catecholamines.

### 3.4. Chronic Pain and Psychiatric Illness

Chronic pain diseases, such as fibromyalgia, may be related to nocturnal “sympathetic overdrive,” and in animal models, increased plasma cortisol and epinephrine levels have been noticed upon long-lasting stress due to chronic pain [27].

Up to 42% of TTS patients suffer from psychiatric illness, and half of those had previously been diagnosed with an affective disorder [3]. Elevated sympathetic activity and reduced catecholamine uptake might be responsible for persistent cardiac sympathetic stimulation, increasing the risk for TTS in these patients, when exposed to stressful situations [28, 29]. Furthermore, the use of selective serotonin reuptake inhibitors (SSRIs) has been associated with a lower survival rate in TTS [30]. In animal models, lithium appears to stimulate catecholamine release from the adrenal gland and increase catecholamine concentration in the plasma [31]. However, lithium seems to reduce plasma epinephrine response upon insulin stimulation in humans [32]. Thus, it is unclear if a long-standing medication with SSRIs and/or lithium plays a role in the development of TTS.

A strong connection between the central nervous system (CNS) and the heart in TTS is highly probable as sympathetic overactivity seems to be an important factor in the development of TTS [17, 33]. Moreover, substantial anatomical alterations in the limbic system have been reported in TTS patients [34]. Decreased cortical thickness in the limbic system might result in less efficient emotional control, predisposing then for TTS. However, this hypothesis need to be confirmed in longitudinal studies [34]. Interestingly, similar brain alterations in the limbic system were found in major depression and bipolar disease when compared that in to healthy controls [35, 36]. These alterations have also been coupled to emotional dysregulation in bipolar disease [36].

**Figure 2:**
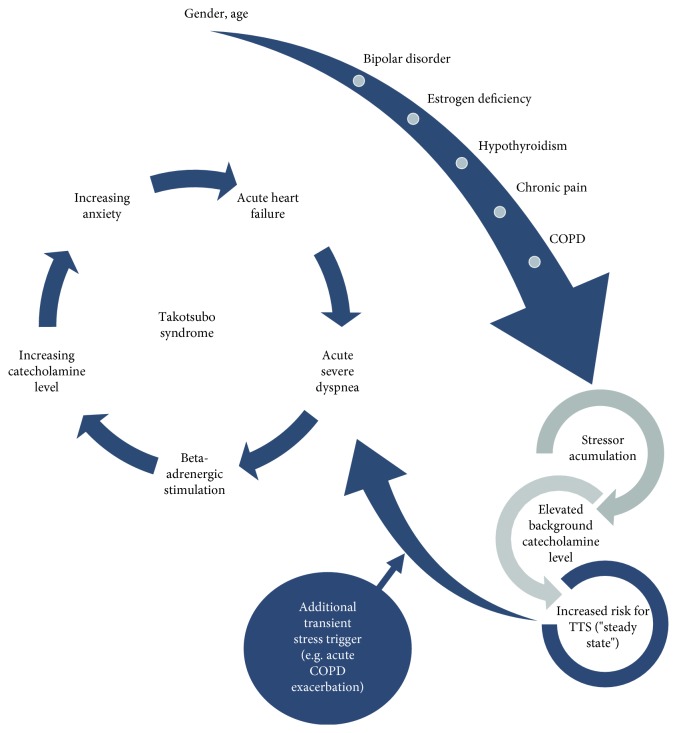
Recurrent Takotsubo syndrome in a 70-year-old female COPD patient. A 70-year-old female patient with several underlying medical and psychiatric conditions presented to the ER in 4 different occasions due to severe acute dyspnea related to acute COPD exacerbation. In all occasions, new ECG abnormalities, elevated cardiac troponin levels, and ECHO-verified acute heart failure were observed. Coronary angiography was performed without signs of coronary stenosis. COPD: chronic obstructive pulmonary disease; ECG: electrocardiogram; ER: emergency room.

Previous to FMC, the patient had a long history of bipolar disorder with several episodes of mania or severe depression. Her last hospitalization due to severe psychiatric illness was registered two years before FMC. There is no data indicating that the patient's background psychiatric illness was acutely aggravated prior to her hospitalizations due to dyspnea. During the 48 months between FMC and the last contact with the ER due to dyspnea, the patient was hospitalized several times due to psychiatric disease, including severe depression and acute stress reaction. Still, none of these episodes was coupled to chest pain or severe dyspnea. Additionally, hospitalizations due to psychiatric illness and dyspnea were registered with a period of several months in between them. This suggests a physical TTS trigger in our patient, in this case acute COPD exacerbation, even if a combination of emotional and physical triggers could also be possible. Interestingly, all the TTS episodes started during the night hours, which also was the time of the day when the patient felt more intensified muscular pain and anxiety. In theory, both conditions, chronical pain and psychiatric illness, might predispose for increased levels of catecholamines and other stress hormones (possibly having an additive effect), which in turn might increase the risk for the development of TTS upon an external trigger. Suffering from both conditions for several years, our patient developed TTS first during her first event of severe dyspnea due to acute COPD exacerbation. This may have been the droplet that caused the beaker to overflow (Figure 2). Here, we suggest an explicatory model in which the patient's previous medical and psychiatric conditions predispose the development of TTS due to chronic increased stress levels with subsequent elevation of systemic catecholamines. Upon additional stressors or triggers, such as in this case severe dyspnea (with consequent hypoxemia, acidosis, and anxiety), the further catecholamine elevation might initiate a cascade of mechanisms resulting in manifest TTS.

### 3.5. Outcome

Although originally considered a less severe condition, TTS is nowadays considered a serious clinical condition with potentially poor prognosis as early and late mortality can be similar to those observed in ACS [37]. Typical type TTS and atypical type TTS appears to have comparable in-hospital mortality and a similar prognosis at long-term follow-up [38]. Additionally, a recent study revealed that patients with TTS secondary to neurological diseases had the worst prognosis [24]. Moreover, patients with emotional stressors showed the most favorable outcome when compared to ACS patients and TTS patients secondary to physical stressors; a novel classification for TTS has also been proposed (InterTAK Classification) based on the type of triggering event [24].

Among others, physical triggers and admission LVEF < 45% are connected to adverse in-hospital outcome [3]. Moreover, a high heart rate is associated with increased mortality in TTS [39]. All these features were observed in our patient. However, common in-hospital complications, such as cardiac arrhythmias, ventricular thrombus, and cardiogenic shock were absent. On the contrary, our patient was fully recovered clinically before leaving the hospital (4 to 16 days, Table 2). Why our patient recovered rapidly is undetermined, but possibly it could be related to the main physical stressor, acute COPD exacerbation, for which there are effective therapeutic management strategies. Besides fatigue and shortness of breath, our patient does not show clinical signs related to cardiac dysfunction. These symptoms are nonspecific and could more likely be caused by her other medical conditions, specially COPD. Curiously, the HEROIC study revealed that post-TTS patients can develop a persistent, long-term heart failure phenotype in which fatigue and shortness of breath are common [40, 41]. The importance of this reported heart failure phenotype in recurrent TTS has to date not been investigated.

## 4. Conclusions

Acute COPD exacerbations might induce TTS in patients with concomitant psychiatric conditions and/or chronic pain. If so, the use of a beta-2 agonist should be limited or avoided in these patients and other treatment options, such as inhaled ipratropium and corticosteroids, should be prioritized. The patients might also have the benefit of an early ECHO if electrocardiographic abnormalities are observed, cardiac troponin levels are elevated, or if chest pain is described. However, “bronchogenic TTS” has been described as an atypical form of TTS, which might lack chest pain as a cardinal symptom [10].

This case report exemplifies the difficulties of the diagnosis of TTS on the onset of acute COPD exacerbation. In order to the prevent recurrence, improve acute treatment, and change the prognosis of “bronchial TTS,” further research and elucidation of the involved mechanisms are demanded. Until then, the recently published InterTAK diagnostic algorithm in TTS is a promising diagnostic tool that, if it had been made available a few years earlier, could have led our patient's medical history towards an early TTS diagnosis with less morbidity and possibly less recurrence.

## Figures and Tables

**Table 1:** Characteristics of a 70-year-old woman presenting with severe dyspnea.

	Diagnosis	Medications	Timeline
Psychiatric history	Bipolar disorder	Lithium Lamotrigine Escitalopram Zopiclone	35 years before FMC Regular contact with psychiatrist

Medical history	Previous ischemic stroke with remaining minute weakness of the right leg	Aspirin Simvastatin	12 years before FMC
	Lithium-induced hypothyroidism	Levothyroxine	20 years before FMC
	Fibromyalgia	Paracetamol	5 years before FMC

Surgical history	Endometrial cancer	Curative hysterectomy with bilateral salpingo-oophorectomy Adjuvant radiochemotherapy	15 years before FMC

Allergies	None		

Family history	Endometrial and colon cancer		

Alcohol use	None		

Nicotine use	Heavy smoker. 35-Pack-year history		

FMC: first contact with the emergency room due to severe dyspnea.

**Table 2:** Clinical presentation of a 70-year-old female COPD patient with recurrent TTS.

	FMC	32 months after FMC	40 months after FMC	40.5 months after FMC	46 months after FMC	48 months after FMC
Symptoms	Severe dyspnea	Dyspnea, exhaustion	Severe dyspnea, cough	Dyspnea, chest pain	Severe dyspnea	Dyspnea
ECG (Figure 1)	T-wave inversion	T-wave inversion	T-wave inversion	Mainly normal	T-wave inversion	Mainly normal
TPNT (ng/L)	53-108-54-14	58-288-285-88	19-200-300-240-54	107-112-52	31-431-521-574-51	42-74-107
NT-pro-BNP (pg/mL)	Normal	2088	1000	Normal	Normal	Normal
CT scan or CR	Normal	Normal	Bilateral pleural effusion	Normal	Normal	Normal
Coronary angiography	Normal	Normal	Normal	None	None	None
ECHO	Normal LVEF 60%	Hypokinesia in the septum and inferolateral wall. LVEF 30%	Global hypokinesia (partially preserved function in the basal wall). LVEF 20%	Normal	Midventricular akinesia but less affected function in apical and basal segments LVEF < 20%	None
ECHO (reviewed images)	Discrete apical hypokinesia	Severe hypokinesia, most prominent midventricular	Severe hypokinesia, most prominent midventricular and apical	Further analysis not possible due poor image quality	As above	None
Days at hospital	7	4	16	5	7	1
Initial diagnose	Myocarditis	Non-Q-wave MI	Myocarditis	Chest pain (UNS)	Midventricular TTC	Dyspnea (UNS)
Diagnose (after review)	Possible TTC	Midventricular TTC	Midventricular TTC	Unclear	Midventricular TTC	Unclear
Follow-up	ECHO: normal	CMR: normal ECHO: normal	ECHO: normal	ECHO: normal	ECHO: normal LVEF Minor basal hypokinesia	CCTA: no significant stenosis

CCTA: cardiac computed tomography angiography; CMR: cardiac magnetic resonance imaging; COPD: chronic obstructive pulmonary disease; CR: chest radiography; ECG: electrocardiogram; ECHO: echocardiography; FMC: first medical contact; LVEF: left ventricular ejection fraction; MI: myocardial infarction; NT-pro-BNP: NT-probrain natriuretic peptide; TPNT: troponin T; TTS: Takotsubo syndrome; UNS: unspecified.
